# How Do Dental Professionals and Students Assess Orthodontic Case Complexity?

**DOI:** 10.1111/eje.13141

**Published:** 2025-06-13

**Authors:** Giulia Semerari, Antonino Peluso, Anthony Carlino, Francesco Moscagiuri, Michele D'Attilio

**Affiliations:** ^1^ Department of Innovative Technologies in Medicine & Dentistry University “G. D'annunzio” Chieti‐Pescara Chieti Italy; ^2^ Department of Human Sciences, Law, and Economics Telematic University Leonardo da Vinci, UNIDAV Chieti Italy

**Keywords:** case complexity perception, diagnostic accuracy, orthodontic diagnosis, orthodontic education

## Abstract

**Background:**

Accurately diagnosing and assessing orthodontic case complexity is essential for treatment planning, estimating therapy duration, and determining required expertise. Various indices, such as the American Board of Orthodontics Discrepancy Index (ABO DI), provide objective evaluations. This study explores how different dental professionals perceive case complexity and compares their assessments to the ABO DI.

**Materials/Methods:**

Thirty pre‐treatment orthodontic records from patients aged 6 to 42 years were classified as mild, moderate, or complex based on DI scores. An online survey was sent to 1289 dental professionals (students, general dentists, orthodontic specialists, and residents), who rated the 35 cases on a complexity scale from 0 (simple) to 10 (complex).

The respondents' rating was then compared with the ABO DI score to assess the perception of orthodontic case complexity.

**Results:**

A total of 131 respondents participated, including 31 students, 68 general dentists, 20 orthodontic specialists, and 12 residents. The sample included 86 men and 45 women, with an average age of 34 years. General dentists tended to overestimate complexity compared to specialists and residents. Specialists perceived cases as more challenging than residents, while students rated cases as more complex than both. Across all groups, 57% of ratings aligned with DI scores, with general dentists achieving the highest agreement (63%). Specialists, residents, and students showed approximately 50% alignment.

**Conclusions:**

Variability in perceived complexity underscores the importance of specialised training for accurate diagnosis. The findings emphasise the need for enhanced orthodontic education for general dentists to improve diagnostic precision and treatment outcomes.

## 1 Introduction

Accurate and comprehensive diagnostic processes are fundamental to establishing effective treatment plans and achieving successful outcomes in medical therapies.

In orthodontics, diagnosing occlusal and skeletal issues, as well as harmful habits, involves medical history, objective examinations (intraoral and extraoral), precise clinical analyses, detailed evaluations of dental casts, radiographic examinations, and cephalometric analyses. For this purpose, auxiliary instrumental examinations such as kinesiography and electromyography are particularly useful [1, 2].

Correct diagnosis also requires clinicians to assess the case's complexity, which informs the effort and skill needed for resolution [3].

The initial complexity evaluation helps identify the necessary level of competence for treating the specific case, to adequately inform the patient about the treatment plan, inherent difficulties, and the required degree of cooperation [4] and to estimate the therapy's duration [5].

DeGuzman et al. in 1995 found a significant association between the perception of malocclusion severity and the difficulty of treatment [6].

There are several indexes of complexity such as the Peer Assessment Rating (PAR) index and the American Board of Orthodontics Discrepancy Index (ABO DI), both validated, which the clinician can use to evaluate the complexity of orthodontic cases.

The application of a complexity index in orthodontic treatment serves several critical functions:

- It determines the level of expertise necessary for managing specific cases [4]
- It provides a standardised method to objectively evaluate the complexity of orthodontic cases.
- It acknowledges the professionalism of practitioners involved in complex treatments [4]
- It enhances the information provided to patients regarding their treatment, including potential challenges and the level of cooperation required [4]

Specifically, the PAR index estimates the deviation of dental malocclusion from normal alignment and physiological occlusion. It evaluates pretreatment and post‐treatment scores to measure the degree of improvement achieved through treatment [7]. While the PAR index is reliable, it does not assess all aspects of malocclusion that need correction for cases evaluated by the American Board of Orthodontics (ABO) [8].

The ABO index is reliable, [9], objective, relatively fast, comprehensive and accurately identifies the complexity of orthodontic cases based on the observation and measurement of orthodontic records such as dental models, cephalometric analysis and panoramic radiography [10].

The ABO DI has been widely used for assessing the complexity of malocclusion. However, few studies have examined its relevance to clinical judgement and its comparison with other indices [11]. Among the limited research, a study demonstrated that the ABO DI is reliable and correlates well with clinical judgement in identifying the severity of malocclusion [12].

The ABO DI analyses: Overjet, overbite, anterior open bite, lateral open bite, crowding, occlusion relationship, lingual posterior crossbite, buccal posterior crossbite, ANB angle, lower incisor to MP (IMPA), and SN‐GoGn angle (SN‐ MP) and other components as aggravating factors of malocclusion like: supernumerary teeth, ankylosis of permanent teeth, anomalous morphology of tooth size and shape, impaction (except 3rd molars) of teeth, missing teeth (except 3rd molars), midline discrepancy, spacing, tooth transposition, skeletal asymmetry (treated non surgically) and additional therapeutic complexities [13].

The higher the score, the greater the complexity of the case [10]. An adequate and successful orthodontic treatment plan must not only rely on a meticulous diagnosis but also be customised for the patient using techniques and orthodontic appliances that are most suited to the resolution of the individual case. Finally, the clinician's ability to identify the complexity of the case is an essential skill to plan and manage the treatment.

Although it is believed that specialised training in the orthodontic field affects the diagnostic decision‐making process and consequently the clinical outcome [14], orthodontic treatments can be performed by both dentists and orthodontic specialists. The ability of non‐specialists to provide orthodontic treatment is often the object of debate and controversy, despite the continuing in‐depth orthodontic training provided by some dentists through training courses [15].

It is considered that individual assessment of the need for orthodontic therapy can be rather subjective and may depend both on the individual experience and on the specialist training obtained [16]. Although orthodontists and general dentists exhibit high levels of agreement regarding the need for orthodontic treatment [16], orthodontists offer superior quality treatment and achieve results in less time compared to general dentists [17].

However, it has emerged from studies that non‐specialised dentists provide 20% to 50% of all orthodontic treatments [18].

Non‐specialised dentists tend to treat a greater number of difficult orthodontic cases in their studies rather than seeking advice from a specialist in orthodontics [19].

It emerged that the use of sequential transparent aligners is one of the most desirable therapeutic devices by non‐specialised dentists [14].

Other studies have investigated the lack of confidence among dental students in orthodontic diagnosis, highlighting the need to enhance university education programmes, particularly by improving students' clinical experience [20].

On the basis of the initial premises, our study collecting a representative sample of dentistry students, dentists, orthodontic residents and specialised orthodontists aims to analyse the perceived orthodontic case complexity by these different groups, to compare it and, later, to relate it with the ABO ID and, therefore, to verify whether or not they have deviated from it.

## 2 Material and Methods

A total of 136 pre‐treatment orthodontic records from patients treated at the Unit of Orthodontics, University of Studies G. d'Annunzio, Chieti were selected for the study. Informed consent was obtained from all study participants prior to their involvement in the research, and the research has been conducted in accordance with the Declaration of Helsinki.

The patient's age ranged from 6 to 42 years, with deciduous, mixed or permanent dentitions who underwent orthodontic treatment without the need for orthognathic surgery.

Orthodontic records excluded from the study were the following:

– twin patients
– incomplete pre‐treatment records
– Patients with craniofacial anomalies
– surgical orthodontic cases
– patients with a history of previous orthodontic treatment (functional, fixed, or aligner)
– Patient with systemic disease affecting temporo‐mandibular joint (ex. juvenile idiopathic arthritis)
– Patient with temporo‐mandibular disorders

**FIGURE 1:**
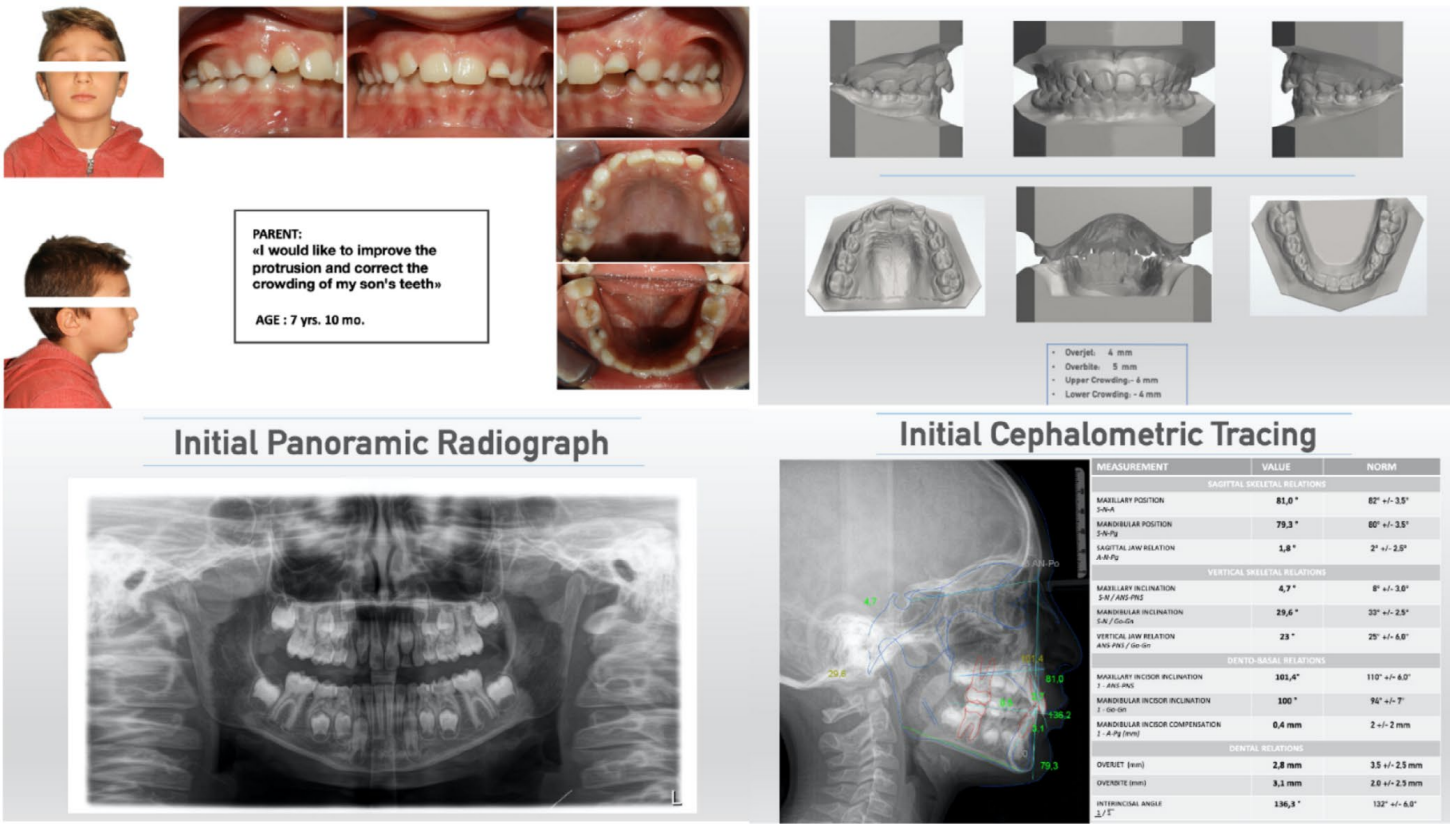
(Upper left) Age, reason for visit, anonymized extra‐oral photos, intra‐oral photos. (Upper right) Digital arch models and overjet value (OJ), overbite value (OB) and upper and lower dental arch crowding. (Lower left) initial orthopantomography. (Lower right) lateral teleradiograph with EBO cephalometric analysis.

Each individual case was presented with the following information [Figure 1]:

– patient age and reason for the visit
– Anonymized extra‐oral photos (front and right side)
– intra‐oral photos (front, left and right side, upper and lower arch)
– digitalized arch models (front, right and left side, upper and lower arch and postero‐front vision)
– clinical values of: overjet, overbite, upper and lower crowding
– initial panoramic radiograph
– latero‐lateral X‐ray with cephalometric analysis

Digital models were obtained by means of intra‐oral scanner Trios‐3shape (3Shape A/S, Copenaghen, Denmark) and cephalometric analysis was performed with the software ZeroBase Orthodontic System (ZeroBase; TIM S.r.l., Puja di Prata, Italy).

For case presentation, the cephalometric track of the European Board of Orthodontics (EBO) was used [21].

Two authors (M.D.A. and F.M.) were calibrated on the calculation of DI scores, and they independently analysed the cases. Only cases with an exact match between the two investigators' DI scores were maintained (30 cases); others were eliminated from the study (106 cases).

A match was defined as both authors classifying the case as mild, moderate or complex.

A total of 30 cases were divided in 3 groups: 10 mild cases (DI, 0–10), 10 moderate cases (DI, 11–20) and 10 complex cases (DI, > 20).

An online survey questionnaire was distributed via email to a total of 1289 recipients, including dental students, general dentists, orthodontic specialists, and orthodontic residents. The questionnaire accepted responses from April 2023 through May 2024, and 138 were fully completed. Incomplete questionnaires were automatically excluded from the analysis.

The initial section of the questionnaire included general questions designed to gather information on:

– age,
– gender,
– qualifications,
– years of experience as dentist or years of education as dental student,
– where orthodontic training was obtained (university programs, specialisation, continuing education courses, master's degree, etc.),
– how university education in orthodontics is rated,
– the types of appliances used in orthodontic practice.

The second section of the questionnaire presented 35 orthodontic cases, consisting of 30 unique cases, 5 of which were duplicates. Participants were asked to rate the orthodontic case complexity on a scale from 0 (simple) to 10 (complex).

The five repeated cases were included to assess the reliability of responses and the level of attention given to completing the questionnaire.

A specific threshold was established to determine the reliability and attention of participants. Those whose responses varied by more than 5 points in the five repeated cases were excluded from the sample to avoid distorting the results. Consequently, the initial sample of 138 participants was reduced to 131, as 7 participants displayed significant inattention.

### 2.1 Statistical Analysis

Statistical analysis was performed using the Prism‐GraphPad software (Graphpad software LLC, San Diego, CA, USA). The data were organised into four groups:

– General dentists (GD)
– Orthodontic specialists (OS)
– Orthodontic residents (OR)
– Dentistry students (DS)

The group of general dentists was then divided into two subgroups

– No‐Trained General Dentists (NTGD), referring to practitioners without any postgraduate education in orthodontics
– Trained General Dentists (TGD), for practitioners who have completed postgraduate master's programmes or courses in orthodontics

The homogeneity of the variances in the samples under analysis was assessed. Depending on the outcome of this test, either the T‐Test or the Welch Test was selected for further analysis.

After conducting the statistical tests, the subjects' assessments of case complexity were compared with the DI score. The subjects' perceptions, initially collected using a quantitative scale from 0 to 10, were converted into a qualitative scale with three categories: “mild,” “medium,” and “complex.” The qualitative categorization was as follows:

– 0 to 3: Mild
– 4 to 6: Medium
– 7 to 10: Complex

Similarly, the DI score results were converted from a quantitative scale to a qualitative scale as follows:

– 0 to 12: Mild
– 13 to 24: Medium
– 25 and above: Complex

## 3 Results

### 3.1 Sample Demographics and Experience Level

The results indicate that the sample group comprises 86 men (65.6%) and 45 women (34.4%), ranging in age from 20 to 68 years, with an average age of 34 years. The participants were categorised into four groups: dentists (68 individuals, 51.9%), orthodontic specialists (20 individuals, 15.3%), orthodontic residents (12 individuals, 9.2%), and dental students (31 individuals, 23.7%).

Regarding experience in the dental field, participants were divided into five groups based on years of experience: 0–5 years, 5–10 years, 10–20 years, 20–30 years, and more than 30 years. The results for each experience range are provided in Table 1.

**TABLE 1:** Years of experience in dental field for the four groups of dental professionals.

	0–5 years	0–5 years (%)	5–10 years	5–10 years (%)	10–20 years	10–20 years (%)	20–30 years	20–30 years (%)	> 30 years	> 30 years (%)
General dentists	36	52.94%	9	13.24%	10	14.71%	4	5.88%	9	13.24%
Orthodontics specialists	1	5.00%	9	45.00%	4	20.00%	4	20.00%	2	10.00%
Orthodontic residents	5	41.67%	5	41.67%	0	0.00%	2	16.67%	0	0.00%
Dental students	11	35.48%	20	64.52%	0	0.00%	0	0.00%	0	0.00%
Total (*n* = 131)	53	40.46%	43	32.82%	14	10.69%	10	7.63%	11	8.40%

Table 1 indicates that 53 subjects (40.436%) have 0–5 years of experience in the dental field, 43 (32.82%) have 5–10 years, 14 (10.69%) have 10–20 years, 10 (7.63%) have 20–30 years, and 11 (8.40%) have over 30 years.

In particular for the dental student group, 64.52% were in the sixth year, while the remaining 35.48% were in the fourth or fifth years of university.

The first two categories (0–5 years and 5–10 years) represent nearly 75% of the total sample (69 out of 131).

This higher proportion in the initial categories can be explained by including university training in the dental field.

Orthodontic training has been categorised as follows:

– Undergraduate degree programmes
– Specialisation
– Master's degrees
– Various orthodontic courses
– Other forms of training, including dental practices and hospital/university orthodontic departments

Among the 131 survey participants, 87 (66.41%) completed an undergraduate degree, 31 (23.66%) specialised in orthodontics, 33 (25.19%) earned a master's degree in orthodontics, 50 (38.17%) attended various orthodontic courses, and 8 (6.11%) received training in other forms, including dental practices or hospital/university orthodontic departments.

Table 2 lists the orthodontic appliances used by each of the four groups.

**TABLE 2:** Orthodontic appliances used by the different groups of dental professionals.

	General dentists (*n* = 68)	Orthodontic specialists (*n* = 20)	Orthodontic residents (*n* = 12)	Dental students (*n* = 31)
Space maintainers	35,29%	65,00%	75,00%	16,13%
Palatal expanders	45,59%	100,00%	100,00%	22,58%
Fixed appliances	50,00%	100,00%	100,00%	19,35%
Functional appliances	50,00%	80,00%	91,67%	16,13%
Extra‐oral appliances	17,65%	55,00%	75,00%	6,45%
aligners	51,47%	100,00%	75,00%	12,90%
Sectional appliance	27,94%	70,00%	58,33%	0,00%
none	35,29%	0,00%	0,00%	70,97%
other	0,00%	20,00%	8,33%	0,00%

The fourth column, representing dental students, is the only group with a high percentage (70.97%) in the “none” section, indicating limited use of orthodontic appliances. Orthodontic specialists and orthodontic residents demonstrate a higher utilisation of all types of appliances compared to dentists. The most commonly used appliances among these groups are palatal expanders, fixed appliances, functional appliances, and clear aligners.

As shown in the table, orthodontic specialists and orthodontic residents use a broader range of appliances than dentists. All orthodontic specialists reported using clear aligners, while 75% of orthodontic residents and 51.47% of dentists indicated they used them.

Participants were asked to assess the quality of their university education in orthodontics on a four‐point qualitative scale (insufficient, sufficient, good, excellent). For the statistic calculation and in order to generate the graph, these qualitative values were converted into quantitative values on a scale from 0 to 100.

**FIGURE 2:**
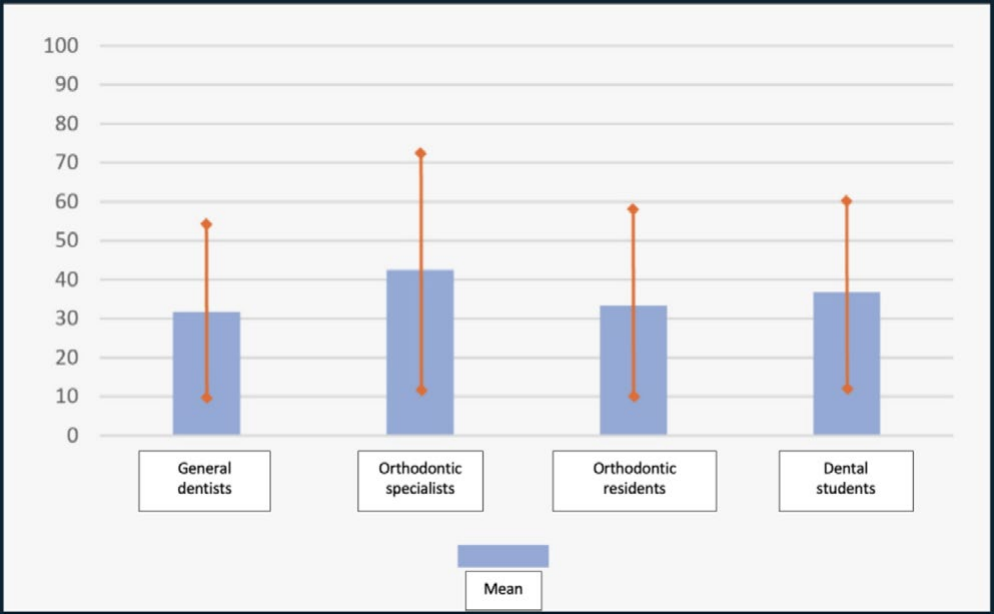
Means and standard deviation of orthodontic education evaluations for each group. Blue bars indicate the means and error bars indicate standard deviation.

Orthodontic specialists rated the quality of their education the highest, with an average score of 42.49 and a standard deviation of 29.91. Orthodontic residents and dentistry students had similar average scores of 33.32 (± 23.43) and 36.68 (± 23.70), respectively. Dentists gave the lowest ratings for the quality of orthodontic education, with an average score of 31.61 (± 22.49). The graph with all the results is shown in Figure 2 with the means and standard deviation of orthodontic education evaluations for each group.

### 3.2 Perception of Case Complexity

In order to investigate this aspect, an analysis has been conducted aimed at comparing subjective perceptions of case complexity across different professional groups. Table 3 presents the ABO DI score alongside participants' rating.

**TABLE 3:** ABO DI score compared with dental professionals score of complexity perception.

	ABO DI	Specialist	Orthodontic resident	General dentist	Student
Mild cases	8.4 ± 2.1	4 ± 1.02	4 ± 0.6	5 ± 0.8	5 ± 1.08
Moderate cases	18.2 ± 1.8	6 ± 1.1	5 ± 1.1	6 ± 0.6	6 ± 0.6
Complex cases	32 ± 6.5	7 ± 0.7	6 ± 0.9	7 ± 0.2	7 ± 0.5

To assess the perceived case complexity, first of all data distribution was assessed with the Kolmogorov–Smirnov test. Due to the lack of normal distribution across all variables, the Welch's test was used for comparisons. The level of significance was set at a *p*‐value of < 0.05. The results of these analyses are detailed in Table 4.

**TABLE 4:** Complexity cases perception for each study group.

Group A	Group B	Mean difference and SEM	95% confidence interval	*p*
General dentist Mean ± SD: 6.06 ± 1.94	Orthodontic specialists Mean ± SD: 5.50 ± 2.45	−0.55 ± 0.11	−0.77 to −0.33	< 0.0001
General dentist Mean ± SD: 6.06 ± 1.94	Orthodontic residents Mean ± SD: 4.94 ± 2.25	−1.11 ± 0.13	−1.37 to −0.85	< 0.0001
General dentist Mean ± SD: 6.06 ± 1.94	Dental student Mean ± SD: 5.75 ± 1.83	−0.3 ± 0.07	−0.45 to −0.15	< 0.0001
Orthodontic specialists Mean ± SD: 5.50 ± 2.45	Orthodontic residents Mean ± SD: 4.94 ± 2.25	−0.55 ± 0.16	−0.87 to 0.16	0.0006
Dental student Mean ± SD: 5.75 ± 1.83	Orthodontic specialists Mean ± SD: 5.50 ± 2.45	−0.2 ± 0.11	−0.48 to −0.01	0.0343
Dental student Mean ± SD: 5.75 ± 1.83	Orthodontic residents Mean ± SD: 4.94 ± 2.25	−0.8 ± 0.13	−1.08 to −0.53	< 0.0001
No‐trained general dentists Mean ± SD: 6.29 ± 1.9	Trained general dentists Mean ± SD: 5.78 ± 1.96	−0.51 ± 0.08	−0.68 to −0.33	< 0.0001

Statistically significant differences were observed across all variables. In particular, dentists reported higher scores than the other three groups, indicating a greater perceived level of difficulty on average. Among the groups, specialists generally perceive cases as more complicated than residents, while students perceive cases as more complex than both specialists and residents. No‐trained general dentists view cases as slightly more complex compared to trained general dentists.

Table 5 shows the degree of agreement between the ABO DI Score and the case complexity perception by the different groups.

**TABLE 5:** Perception of the cases complexity detected by the subjects of the four groups compared with the DI score.

	General dentists	Orthodontic residents	Orthodontic specialists	Dental students	Total mean
Not according with DI score	37%	50%	53%	47%	43%
According with DI score	63%	50%	47%	53%	57%

On average, across all four groups, 57% of subjects align with DI score, while 43% do not. Among the four groups, dentists have the highest percentage (63%) of agreement between their perceptions and the DI. In contrast, specialists, postgraduates, and students each have around a 50% alignment with the DI.

Table 6 provides a comparison between trained general dentists and non‐trained general dentists. The comparison indicates no significant differences between the two subgroups in terms of their perception of case complexity and alignment with the DI score.

**TABLE 6:** Perceptions of the cases complexity between the two subgroups compared with the DI score.

	Trained general dentists	Not trained general dentists
Not according with DI score	43%	40%
According with DI score	57%	60%

## 4 Discussion

The study aimed to achieve three main objectives. First, to identify overarching themes enabling us to gather information about our sample, including their years of experience in dentistry, level of orthodontic training, utilisation of orthodontic devices in clinical practice, and the evaluation of orthodontic training received at universities. Second, to compare perceptions among four distinct groups to ascertain potential differences. Third, to evaluate whether the perceived complexity of cases differed between the groups and aligned with the objective complexity assessments provided by the DI.

Our findings reveal significant differences in the use of orthodontic devices across different professional groups. General dentists, orthodontic residents, and specialists demonstrate a consistent use of various orthodontic devices; however, specialists and residents employ them more frequently than general dentists. Notably, all orthodontic specialists utilise clear aligners, whereas 75% of orthodontic residents and 51.47% of general dentists employ them. This prevalence among general dentists may stem from aligner treatment plans often being directly supplied by manufacturers, enabling even those with limited orthodontic expertise to undertake such treatments.

In contrast, dental students exhibit the lowest device utilisation rates, with a substantial 70.97% reporting non‐use. This could be attributed to limited exposure to orthodontic departments during university education. Alarmingly, this trend is more pronounced in our study compared to findings from a study at the University of Texas [22], indicating potentially inadequate clinical exposure in Italian universities, particularly in orthodontic practice.

Specialists and residents demonstrate versatile use of orthodontic appliances, reflecting their ability to tailor treatment plans to individual patient needs. However, perceptions of undergraduate orthodontic training in all groups were poor, consistent with findings from previous research [22].

Increasing clinical exposure and practical training could enhance the efficacy of orthodontic education.

On average, orthodontic specialists have evaluated orthodontic training in the university in a better way than residents; this could be indicative of a current decline in the quality of specialised orthodontics programmes compared to previous years.

Regarding perceptions of case complexity, dentists and students tend to perceive cases as more complex, likely due to varying levels of orthodontic training. Specialists, on average, perceive cases as more complex than residents, indicative of their greater experience and critical assessment skills. Notably, dentists exhibit a higher alignment with DI scores compared to other groups, suggesting a proficiency in identifying case complexities despite lacking orthodontic specialisation.

Approximately 75% of participants have less than 10 years of work experience, potentially influencing their perceptions of case complexity compared to the DI scores, as perceived needs for orthodontic treatment are affected by increased experience and skills [23].

Although there is a tendency for participants to overestimate case complexity, this heightened awareness could lead to more meticulous treatment planning and execution.

Our study differs from other studies in the literature [22] in several key aspects

– Differences in the results obtained
– The competencies of general dentists were evaluated more comprehensively by dividing this group into those who had completed private training programmes and those who had never participated in such programmes, allowing for a more detailed analysis and assessment of this group;
– The number of cases assessed by the participants was larger, enabling a more in‐depth evaluation.
– The number of repeated cases to assess attention and reliability was higher.
– The cephalometric analysis used in this study was the EBO cephalometric analysis.
– The geographic area of focus was different from previous studies, representing a region that had not been analysed before.

It's crucial to acknowledge the subjective nature of complexity assessments compared to the objective DI scores, which may account for disparities observed in perceptions.

One limitation of the study is the unequal response across the various groups. General dentists represent more than half of the total sample (51.9%), while the remaining is represented by specialists (15.3%), residents (9.2%) and students (23.7%).

This imbalance could impact results comparability and generalisability. Future studies with more balanced samples are recommended to extend these results.

## 5 Conclusion

General dentists demonstrate perceptions of case complexity most aligned with DI assessments, indicating a certain level of proficiency despite lacking specialised training. However, successful treatment outcomes rely on various factors beyond mere perception, including adept treatment planning and the utilisation of different orthodontic techniques. Thus, further specialisation in orthodontics is advocated to optimise patient care. The findings underscore the importance of enhancing orthodontic education and clinical exposure to improve treatment outcomes.

## Consent

Informed consent was obtained from all subjects involved in the study and the research has been conducted in accordance with Declaration of Helsinki. Consent to images publishing was obtained from the patient's parent.

## Conflicts of Interest

The authors declare no conflicts of interest.

## Data Availability

Data are available at the following link: https://data.mendeley.com/preview/rhvkpmh4cb?a=92da1c20‐24fd‐4a98‐b0d0‐6cd14e2d9a7c.

## References

[1] E. Møller and M. Bakke , “Occlusal Harmony and Disharmony: Frauds in Clinical Dentistry?,” International Dental Journal 38, no. 1 (1988): 7–18. 3290115

[2] F. M. Migliore , L. Breda , E. Di Maria , F. Battestini , B. Di Carlo , and M. D'Attilio , “Clinical and Instrumental Temporomandibular Joint Evaluation in Children and Adolescents With Juvenile Idiopathic Arthritis: A Medium‐Term Follow‐Up,” Applied Sciences 13, no. 24 (2023): 13036.

[3] K. Bergström and A. Halling , “Comparison of Three Indices in Evaluation of Orthodontic Treatment Outcome,” Acta Odontologica Scandinavica 55, no. 1 (1997): 36–43, 10.3109/00016359709091939. 9083574

[4] S. K. Llewellyn , A. M. Hamdan , and W. P. Rock , “An Index of Orthodontic Treatment Complexity,” European Journal of Orthodontics 29, no. 2 (2007): 186–192. 17229790 10.1093/ejo/cjl080

[5] D. Aljehani and H. A. Baeshen , “Effectiveness of the American Board of Orthodontics Discrepancy Index in Predicting Treatment Time,” Journal of Contemporary Dental Practice 19, no. 6 (2018): 647–650. 29959290

[6] L. DeGuzman , D. Bahiraei , K. W. Vig , P. S. Vig , R. J. Weyant , and K. O'Brien , “The Validation of the Peer Assessment Rating Index for Malocclusion Severity and Treatment Difficulty,” American Journal of Orthodontics and Dentofacial Orthopedics 107, no. 2 (1995): 172–176. 7847276 10.1016/s0889-5406(95)70133-8

[7] S. Richmond , W. C. Shaw , K. D. O'Brien , et al., “The Development of the PAR Index (Peer Assessment Rating): Reliability and Validity,” European Journal of Orthodontics 14, no. 2 (1992): 125–139. 1582457 10.1093/ejo/14.2.125

[8] J. S. Casko , J. L. Vaden , V. G. Kokich , et al., “Objective Grading System for Dental Casts and Panoramic Radiographs. American Board of Orthodontics,” American Journal of Orthodontics and Dentofacial Orthopedics 114, no. 5 (1998): 589–599. 9810056 10.1016/s0889-5406(98)70179-9

[9] R. M. Pulfer , C. T. Drake , G. Maupome , G. J. Eckert , and W. E. Roberts , “The Association of Malocclusion Complexity and Orthodontic Treatment Outcomes,” Angle Orthodontist 79, no. 3 (2009): 468–472. 19413388 10.2319/042308-227.1

[10] T. J. Cangialosi , M. L. Riolo , S. E. Owens, Jr. , et al., “The ABO Discrepancy Index: A Measure of Case Complexity,” American Journal of Orthodontics and Dentofacial Orthopedics 125, no. 3 (2004): 270–278. 15014402 10.1016/j.ajodo.2004.01.005

[11] K. B. Reagin , “The American Board of Orthodontics Discrepancy Index: An Evaluation of Its Validity,” American Journal of Orthodontics and Dentofacial Orthopedics 130 (2006): 185–191.

[12] S. Liu , H. Oh , D. W. Chambers , S. Baumrind , and T. Xu , “Validity of the American Board of Orthodontics Discrepancy Index and the Peer Assessment Rating Index for Comprehensive Evaluation of Malocclusion Severity,” Orthodontics & Craniofacial Research 20, no. 3 (2017): 140–145. 28670875 10.1111/ocr.12195

[13] American Board of Orthodontics , “The ABO Discrepancy Index (DI) A Measure of Case Complexity,” https://www.americanboardortho.com/media/1189/discrepancy_index_scoring_system.pdf. 10.1016/j.ajodo.2004.01.005 15014402

[14] S. Akyalcin , “Specialist Training Influences the Ability to Recognise Orthodontic Case Complexity,” Journal of Orthodontics 46, no. 1_suppl (2019): 35–38. 30966868 10.1177/1465312519842883

[15] R. J. Smith , “General Practitioners and Orthodontics,” American Journal of Orthodontics and Dentofacial Orthopedics 92, no. 2 (1987): 169–172. 3475971 10.1016/0889-5406(87)90372-6

[16] N. W. Berk , H. D. Bush , J. Cavalier , et al., “Perception of Orthodontic Treatment Need: Opinion Comparisons of Orthodontists, Pediatric Dentists, and General Practitioners,” Journal of Orthodontics 29, no. 4 (2002): 287–291. 12444269 10.1093/ortho/29.4.287

[17] L. S. Marques , N. Freitas Junior , L. J. Pereira , and M. L. Ramos‐Jorge , “Quality of Orthodontic Treatment Performed by Orthodontists and General Dentists,” Angle Orthodontist 82, no. 1 (2012): 102–106. 21806465 10.2319/061311-389.1 PMC8881032

[18] R. M. Jacobs , S. E. Bishara , and J. R. Jakobsen , “Profiling Providers of Orthodontic Services in General Dental Practice,” American Journal of Orthodontics and Dentofacial Orthopedics 99, no. 3 (1991): 269–275. 1998302 10.1016/0889-5406(91)70008-K

[19] A. P. Batarse , J. D. English , G. N. Frey , J. M. Piazza , and S. Akyalcin , “Referral Patterns of Pediatric Dentists and General Practitioners to Orthodontists Based on Case Complexity,” American Journal of Orthodontics and Dentofacial Orthopedics 156, no. 1 (2019): 61–66. 31256840 10.1016/j.ajodo.2018.07.025

[20] H. M. Ismail , A. A. Abdulrahman , and A. I. Ibrahim , “Evaluation of Dental Students and Alumni's Confidence Level in Orthodontic Diagnosis and Treatment‐Planning: A Qualitative Study,” Journal of Dental Education 89, no. 1 (2025): 90–97. 39072737 10.1002/jdd.13679

[21] “European Orthodontic Society,” https://eoseurope.org/wp‐content/uploads/2021/05/appendix_1.pdf, p.16.

[22] E. M. Heath , J. D. English , C. D. Johnson , E. B. Swearingen , and S. Akyalcin , “Perceptions of Orthodontic Case Complexity Among Orthodontists, General Practitioners, Orthodontic Residents, and Dental Students,” American Journal of Orthodontics and Dentofacial Orthopedics 151, no. 2 (2017): 335–341. 28153163 10.1016/j.ajodo.2016.06.045

[23] S. Kuroda , A. Fuji , M. Sugie , et al., “Relationship Between Orthodontic Expertise and Perception of Treatment Needs for Maxillary Protrusion: Comparison of Dental Students, Residents, and Orthodontists,” American Journal of Orthodontics and Dentofacial Orthopedics 137, no. 3 (2010): 340–345. 20197170 10.1016/j.ajodo.2008.04.029

